# Investigation of the carbon dioxide adsorption behavior and the heterogeneous catalytic efficiency of a novel Ni-MOF with nitrogen-rich channels†

**DOI:** 10.1039/d0ra05233g

**Published:** 2020-08-12

**Authors:** Sima Aryanejad, Naser Valipour Motlagh

**Affiliations:** Department of Chemistry, Faculty of Science, University of Birjand Birjand Iran n.valipour.m@birjand.ac.ir nasservalipoormotlagh@gmail.com +98-5632202515 +98-9153636841

## Abstract

MOFs have attracted remarkable attention as solid sorbents in CO_2_ capture processes for their low-energy post-combustion. In this paper, a new Ni-based MOF, Ni_4_(TATB)_1.5_(EtO)_3.5_(NEt_3_)_4_, was synthesized and characterized using various physicochemical techniques. The efficiency of the as-prepared Ni-MOF as a solid sorbent for CO_2_ capture was investigated, and acceptable adsorption was exhibited. Furthermore, this Ni-MMOF was used as a catalyst in toluene selective oxidation, for eliminating a volatile organic compound, with *tert*-butyl hydroperoxide as an oxidant in the absence of organic solvents. The obtained results indicated that Ni-MOF has good catalytic activity and could be reused three times without considerable loss of its catalytic activity. This study highlights the great potential of developing MOFs to achieve green chemistry goals with the removal of hazardous liquid and gas compounds such as toluene and CO_2_.

## Abstract 2

MOFs have attracted remarkable attention as solid sorbents in CO_2_ capture processes for their low-energy post-combustion.

## Graphical abstract

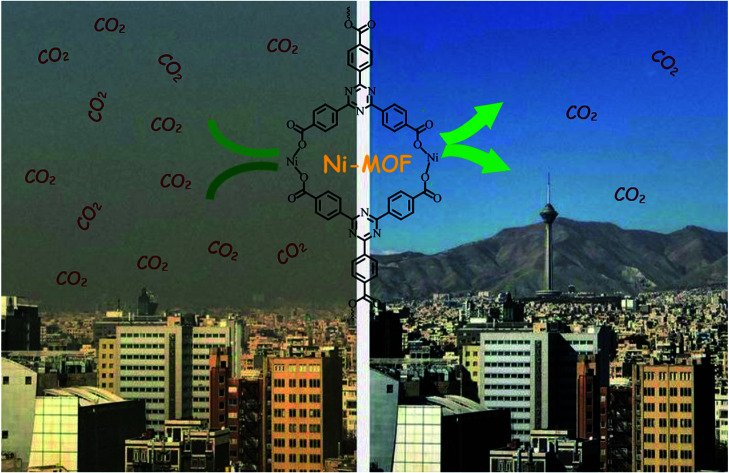

## Introduction

The explosive growth of energy consumption due to anthropogenic activities has been associated with increasing CO_2_ concentration. CO_2_ is the primary contributor to the greenhouse gas effect and the most important perpetrator of global climate change.^1,2^ As a subject of public concern, there is an urgent requirement to reduce the amount of CO_2_ for sustainable development and environmental protection. Carbon capture and storage (CCS) is one of the most hopeful approaches to diminish this issue.^3^ Three main technologies, including cryogenic distillation, absorption, and adsorption, have been employed for CO_2_ capture. Among them, adsorption is a promising technique because it is energetically efficient and economically competitive.^4–6^ Porous materials such as zeolites,^7^ carbons,^8^ porous aromatic frameworks,^9^ and calcium oxide^10^ were previously examined for CO_2_ capture; however, they had the disadvantages of being insufficiently selective or difficult regeneration. Thus, there is a continuous quest for better adsorbents with improved efficiency.

Emerging metal–organic frameworks (MOFs) for the last decades, as a new class of porous materials, have been receiving considerable attention because of their tunable porosities and structures and a wide range of potential applications.^11^ Among their many applications, CO_2_ capture stands out as specific challenges that the field of MOFs is well-prepared to address. Particularly, the reticular chemistry of MOFs has been grown to the point that a chemist can systematically and rationally modulate the interplay between the structure of MOF and the desired properties.^12^ The previous reports exhibited that the incorporation of some functional groups, *e.g.* unsaturated metal sites, heteroatoms and non-metallic functional groups of SBUs, enhance the affinity of the framework towards selective CO_2_ capture.^13^ Of these, heteroatoms incorporation within the backbone has indicated excellent promise for providing strong interactions with CO_2_.^14,15^

With these concepts for structural design in hand, a novel Ni-MOF was designed and synthesized using a rigid trigonal planar ligand, 4,4′,4′′-*s*-triazine-2,4,6-triyl-tribenzoic acid (H_3_TATB), which contains two different functional groups, the carboxylate groups will form neutral MOFs, and the imine groups can act as secondary functional groups in the pores. After full characterization of the synthesized Ni-MOF (named as UoB-20), its adsorption behavior for CO_2_ was investigated that the results revealed the acceptable activity.

The removal of volatile organic compounds (VOCs) has been the main trend because of hazardous effects on human health and the environment. Whereas, the potential of them to transform into valuable and harmless chemicals is usually ignored.^16,17^ Toluene is one of the toxic and carcinogen VOCs that its oxidation to useful chemical products *i.e.*, benzyl alcohol, benzaldehyde, and benzoic acid is extremely attractive. Unfortunately, due to the high activation of C–H bonds, toluene oxidation reaction usually performs in harsh conditions such as high oxygen pressure, high temperature, or the presence of additives.^18,19^ Also, another problem associated with the toluene oxidation processes is the uncontrollable over-oxidation of products and convert to CO_2_, which is the main problem in them. Therefore, there is the main challenge to control the toluene oxidation reaction to produce desirable products without additive under mild conditions. To overcome this issue, it is necessary to introduce a heterogeneous catalyst without the above disadvantages. MOFs can be used as efficient heterogeneous catalysts, due to having polymeric nature and organic–inorganic hybrid composition, which make them good recyclable and reusable catalysts.

With the objective of the catalytic activity of Ni-MOF for producing fine chemicals from the transformation of hazardous compounds, its efficiency in the toluene oxidation to benzaldehyde was investigated. However, to the best of our knowledge, there has rarely been reported on oxidation reactions breaking chemically inert C–H bonds using MOFs as catalyst under mild conditions.

## Experimental

### Materials

All the chemicals and solvents in the experiments were analytical grade and used as received without further purification. Cyanuric chloride (C_3_N_3_Cl_3_), nickel acetate (Ni(OAc)_2_·4H_2_O), acetic anhydride ((CH_3_CO)_2_O), and toluene were provided Sigma-Aldrich Company. Anhydrous aluminum chloride (AlCl_3_), acetic acid (HOAc), and chrome oxide (CrO_3_) were purchased from Merck Company. 4,4′,4′′-(1,3,5-Triazine-2,4,6-triyl)tribenzoic acid (H_3_TATB) linker was synthesized according to previously published methods^20^ (for further information see ESI†).

### Ni-MOF synthesis

H_3_TATB (1 mmol, 0.452 g) linker was dissolved in a DMF (21 mL) by adding triethylamine (4 mL) to form a clear solution. Then, at ambient temperature. Meanwhile, Ni(OAc)_2_·4H_2_O (3 mmol, 0.746 g) was added to deionized water (90 mL) and ethanol (12 mL) for the same time. Subsequently, the Ni(OAc)_2_·4H_2_O solution was gradually added into the H_3_TATB solution and stirred at room temperature for 30 minutes. Then, the mixed solution was kept in the same condition for overnight. After that, the solid product was filtered off and washed with water and ethanol three times. Finally, the synthesized framework, namely as UoB-20 (University of Birjand), was dried at 100 °C in a vacuum oven for 10 hours.

### Characterization

The X-ray diffraction (XRD) pattern was recorded using Philips PW 1730/10 instrument with Cu Kα radiation in the 2*θ* range of 5–50°. X-ray photoelectron spectra (XPS) measurements were carried out by a Thermo Scientific with monochromatic Al Kα (1486.6 eV) at 15 kV and 10 mA. Thermogravimetric analysis (TGA) was obtained from SDT-Q600 between 30 and 710 °C with a heating rate of 10 °C min^−1^ under argon gas. Inductively coupled plasma atomic emission spectroscopy (ICP-AES) analysis was conducted on OPTIMA 7300DV to determine the metal content. Elemental analyses for C, H, and N were obtained IsoPrime100 Elemental Analyser. Infrared (IR) samples were prepared as KBr pellets, and spectra were recorded in the range of 400–4000 cm^−1^ using a NICOLET system. Transmission Electron Microscope (TEM) analysis was performed on a TEM Philips EM 208S. Scanning electron microscopy (SEM) was obtained SEM FEI Quanta 200. Gas sorption measurements were performed a MicroActive for TriStar II Plus. Before the measurement, UoB-20 was degassed under vacuum at 140 °C for 10 h. The surface areas and pore size distributions were determined by the BET and BJH method, respectively. NMR experiments were performed on a Bruker UltraShield™ spectrometer operating at 300 and 50 MHz for proton and carbon, respectively. The chemical shifts are expressed in ppm relative to tetramethylsilane as the internal reference.

### Catalytic activity test

For liquid oxidation of toluene, toluene (1 mmol, 9.42 mL) and catalyst (2.5 mol%) were performed into two necks round bottom flask under a mechanical stirrer. The reaction mixture was heated at 75 °C in the oil bath, and then *tert*-hydrogen peroxide (3 eq.) was added dropwise. The mixture remained at the set temperature for 3 h. The reaction progress was monitored by Thin-Layer Chromatography (TLC). The recyclability of catalyst was performed as the same reaction conditions. At the end of the reaction, catalyst was isolated from the cooled mixture *via* simple centrifugation, thoroughly washed with ethanol several times, dried at 100 °C for 10 h, and then reused in the next cycle.

## Result and discussion

### Ni-MOF synthesis and characterization

**Scheme 1:**
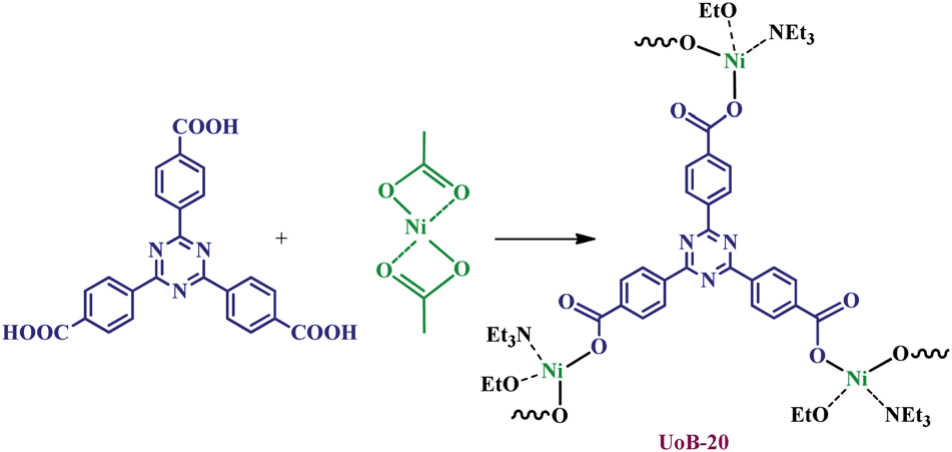
Schematic illustration of synthesis and chemical structure of UoB-20.

The incorporation of chemically available Lewis basic sites into the channels of MOFs is a great challenge. So, it is very significant to develop novel functional porous materials for various applications, for example, selective CO_2_ adsorption and heterogeneous catalysis.^21–24^ Several new approaches were reported for generation available Lewis basic sites inside MOF channels.^25^ Most of the reported methods rely on employing multi-topic bridging ligands containing Lewis basic sites, which cannot easily be coordinated to metal ions during the synthesis of MOF. Also, the produce of intact 3° amine-based Lewis basic sites is rather challenging due to their high basicity. In order to achieve this aim, we have focused on the synthesis of the functional MOF by H_3_TATB, as a 3° amine-based Lewis basic sites, through the simple procedure, which described in the Experimental section (Scheme 1).

**Fig. 1:**
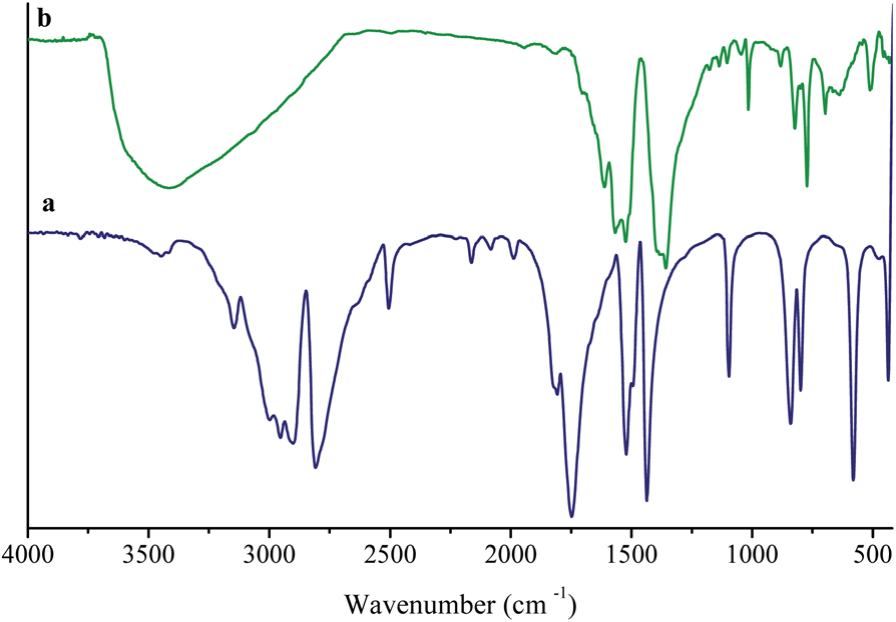
FTIR spectra of (a) H_3_TATB; (b) UoB-20.

The coordination mode of H_3_TATB to Ni^2+^ for the MOF was revealed by the FT-IR spectrum of the as-prepared product. The H_3_TATB, as a strong coordinated ligand, has various coordinate modes, such as unidentate, chelating bidentate, and bridging bidentate. For easy comparison, the FT-IR spectra of the H_3_TATB and Ni-based MOF were presented in Fig. 1. The FT-IR spectrum of aromatic carboxylic acid has been investigated considerably, and all absorption peaks were assigned to the corresponding vibrations.^26^ As shown in Fig. 1a, the peaks at 1716 and 1065 cm^−1^ were attributed to the *ν*(C=O) and *δ*(O–H) stretching vibrations of carboxylic acid groups, respectively. Two distinct absorption peaks at 1490 and 1405 cm^−1^ were attributed to the aromatic ring of N-containing and the side rings. By contrast, the peak of C=O at 1716 cm^−1^ disappears, which detects the carboxyl group coordinated to Ni. The bands at 1623 and 1361 cm^−1^ were assigned to the asymmetric and symmetric stretching modes of the coordinated carboxylate group, respectively. The broad band centered at 3413 cm^−1^ from H_2_O implies the coordinated water and hydrogen-bonding in UoB-20. Furthermore, the coordinate modes of carboxylate group to metal ion were determined by the wavenumber difference (Δ*υ*) between *υ*_as_ and *υ*_s_. The difference between asymmetric and symmetric stretching of COO^−^ anion is 651 cm^−1^, which is greater than 262 cm^−1^ in UoB-20, indicating that the COO^−^ of H_3_TATB coordinated to Ni^2+^ in a bidentate mode.

**Fig. 2:**
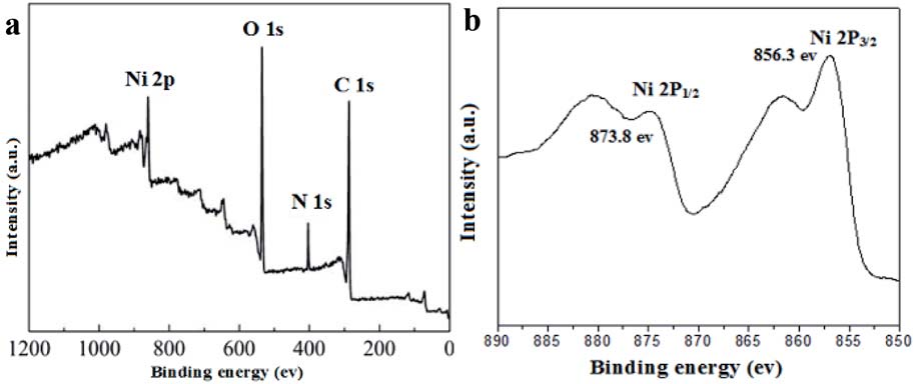
(a) XPS spectra of UoB-20; (b) Ni 2p spectra of UoB-20.

XPS analytical technique was performed to gain further detailed insight into the chemical composition and the chemical valence states of nickel in the UoB-20. The main peaks related to Ni 2p, C 1s, N 1s and O 1s in the full spectrum of UoB-20 proved the existence of Ni, O, and C elements (Fig. 2a). XPS spectrum of Ni 2p exhibited two peaks at 856.3 eV for Ni 2p_3/2_ and 873.8 eV for Ni 2p_1/2_ with a spin-energy separation of 17.5 eV (Fig. 2b). The results well confirmed the presence form of nickel ions is Ni(ii) in the UoB-20, which are in good agreement with the XPS data known for Ni(ii).^20,21^

To clarify the composition of UoB-20, the as-prepared product was further studied by element analysis (EA). EA (%) result found as follows: C, 68.17; N, 10.37; Ni, 10.02. According to electrical neutrality supposition and the EA results, the probable molecular formula was supposed to be Ni_4_(TATB)_1.5_(EtO)_3.5_(NEt_3_)_4_. The calculated EA% (C, 68.13; N, 10.08; Ni, 9.49) based on the proposed formula matched very well with the determined value.

**Fig. 3:**
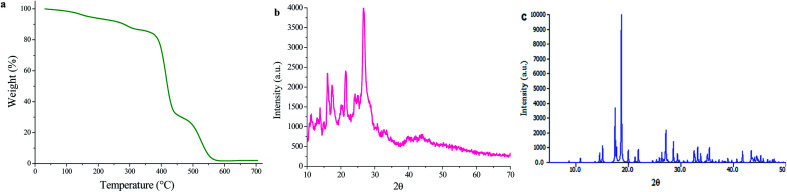
(a) TGA curve; (b) XRD pattern of UoB-20, (c) XRD pattern of Ni-BTC.

TG analysis of UoB-20 was performed under an argon atmosphere at a heating rate of 10 °C min^−1^ to evaluate its thermal stability (Fig. 3a). It is to note that the losing weight (13.57%) was observed below 330 °C, indicating the removal of coordinated ethoxy groups in the UoB-20. This amount compatible with well the calculated value based on the proposed formula (13.3%). The TG curve shows a considerable loss weight between 370 °C and 570 °C for the decomposition of UoB-20 to NiO.

The PXRD pattern of UoB-20 is presented in Fig. 3b, and its pattern shows intense and clear peaks in 16.12, 21.31, and 26.66 2*θ*. The presence of the sharp peaks confirmed the synthesized MOF has high crystallinity. According to the size of the crystals was the smaller than the adequate size for single-crystal determination, the PXRD of UoB-20 was compared to the reported XRD of the previous studies. The overall XRD pattern of the UoB-20 is in good agreement with the Ni-BTC pattern (Fig. 3c), as reported in the literature,^22^ indicating that both of them (UoB-20 & Ni-BTC) are isostructural.

**Fig. 4:**
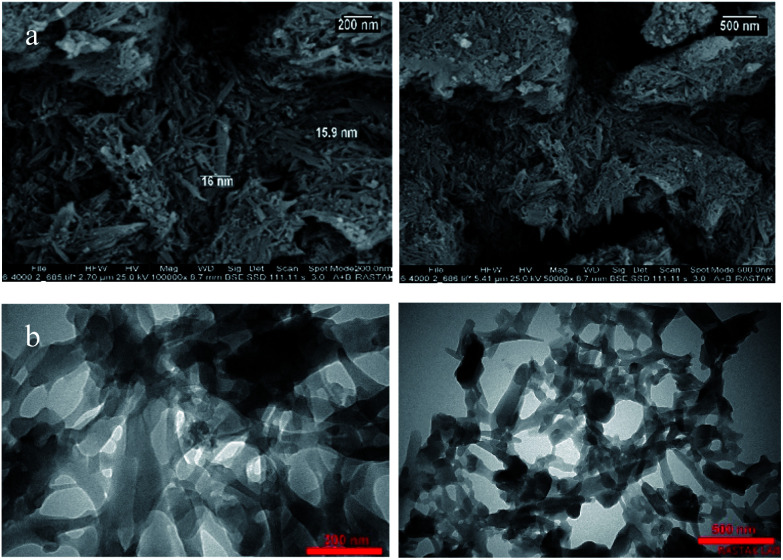
(a) SEM; (b) TEM images of UoB-20.

The morphology and microstructure of the as-prepared product were investigated by SEM and TEM, respectively. The panoramic view in Fig. 4 exhibits that UoB-20 consists of nano-rods in high quantity. The sizes of UoB-20 rods are relatively uniform with the scale of nanometers. Based on the SEM images, the size of UoB-20 nano-rods were about 16 nm without the uniform length. TEM image (Fig. 4b) was obtained to gain further insight into the architecture of UoB-20, which displayed the as-prepared ultrathin nano-rods with nano-scale size. It should be noted that this result is consistent with the result of the SEM image. Furthermore, the TEM image revealed no amorphous component in the synthesized nano-size.

**Fig. 5:**
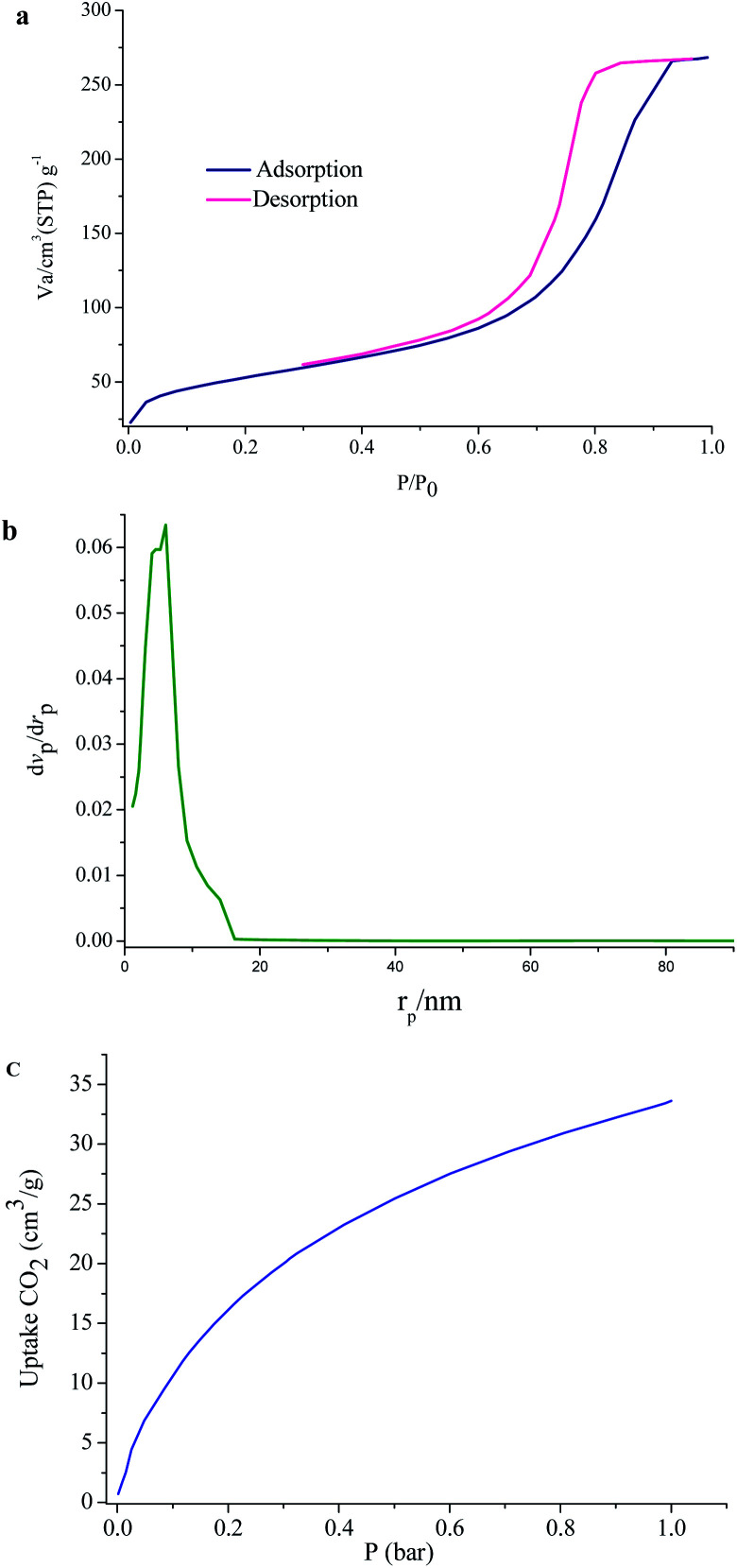
(a) N_2_ adsorption–desorption isotherms; (b) pore width of UoB-20; (c) CO_2_ adsorption at 273 K in UoB-20.

To evaluate the porous nature of UoB-20, Brunauer–Emmett–Teller (BET) gas-sorption measurements were carried out. The N_2_ adsorption–desorption isotherms at 77 K and the corresponding Barrett–Joyner–Halenda (BJH) pore size distribution plot of as-prepared UoB-20 are displayed in Fig. 5. The results exhibited a type IV isotherm with an H_2_ hysteresis loop, based on the IUPAC definition, which revealed the mesoporous structure of UoB-20 (Fig. 5a). The distribution curve of the pore diameter, calculated by the BJH method, exhibited that the dominant mesopores are centered approximately in the range of 3–11 nm with a peak maximum of 6 nm (Fig. 5b). Also, the BET surface area and the pore volume was calculated to be 181 cm^2^ g^−1^ and 0.41 cm^3^ g^−1^, respectively.

### CO_2_ adsorption

MOFs have exhibited the potential to address the world's energy and environmental problems, especially as solid adsorption for CO_2_. In this regard, the ability of UoB-20 was investigated in the CO_2_ adsorption field. The low-pressure adsorption of CO_2_ was studied at 273 K and the related isotherm was plotted in Fig. 5c. The result shows a typical type-I isotherm, which a steep rise at the very low-pressure region. The adsorbed CO_2_ amount for UoB-20 was 33 cm^3^ g^−1^ (1.7 mmol g^−1^). It should be noted that most MOFs exhibit dissatisfactory CO_2_ adsorption capacities at low pressures, which are the realistic pressure of CO_2_ emission. Based on previous research, the incorporation of heteroatoms, especially high polarity of them or with a nucleophilic nature, within the framework, have exhibited a strong interactions with CO_2_.^13^ The CO_2_ capture properties of organic linker with a range of aromatic, primary, secondary and tertiary amines was confirmed in the literature.^23^ Therefore, it can be concluded that the relatively desirable CO_2_ adsorption for UoB-20 is due to the incorporation of the high concentration of 3° amine-based Lewis basic sites in the framework.

To further confirm the efficiency of UoB-20, the obtained result was compared to the previously reported literature (Table 1). The result of the comparison table revealed that UoB-20 has an acceptable CO_2_ adsorption capacity, despite the lower surface area compared to the listed MOFs in the table. It can also be due to the presence of 3° amine-based Lewis basic sites in UoB-20. Finding the positive effect of 3° amine-based Lewis basic sites in the framework for CO_2_ adsorption capacity can be useful for future research in developing excellent CO_2_-absorbing materials.

**Table 1:** CO_2_ adsorption capacities of some adsorbents at 1 bar and 273 K

Adsorbent	Langmuir surface area (m^2^ g^−1^)	Adsorption capacity (mmol g^−1^)	Ref.
UiO-66-CF_3_	799	1.53	24
UiO-66-(COOH)_2_	217	1.70	24
UiO-66-Br_2_	339	1.40	24
NH_2_-MIL-53	1100	1.50	25
{[Zn_2.66_O_0.66_(L)_2_]·2H_2_O}_*n*_^a^	780	1.44	26
[Zn_2_(DMF)(py-CF_3_)_2_]_*n*_	390	1.31	27
VPI-100 (Ni)	612	1.25	28
VPI-100 (Cu)	398	1.44	28
TKL-104 (Ni-tpt)^b^	1131	1.45	29
UoB-20	181	1.44	This work

a L: 2,2′-bis-trifluoromethyl-biphenyl-4,4′-dicarboxylic acid.

b tpt: 2,4,6-tri(4-pyridinyl)-1,3,5-triazine.

### The catalytic activity of UoB-20 towards toluene selective oxidation

**Scheme 2:**
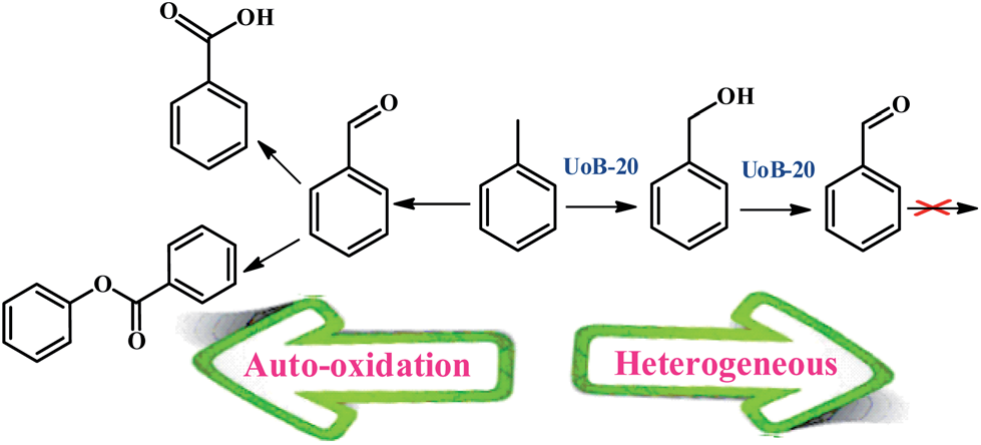
Toluene oxidation reaction with UoB-20 as a heterogeneous catalyst.

Several reports have been presented for the toluene oxidation reaction until now. Although many of these reports are suitable for laboratory synthesis, they are not effective for industrial-scale fabrication due to required difficult conditions of reaction. For instance, most of the reported reactions were performed at high reaction temperatures above 100 °C. Therefore, supplying more simple procedures with available and usual equipment at lower temperatures are required to develop toluene oxidation. Accordingly, the main aim of this study was placed in simplifying the toluene selective oxidation reusable and efficient catalyst by a novel MOF with economical and environmentally friendly conditions at lower temperature (Scheme 2).

To select the best conditions for oxidation of toluene, model research was performed with toluene in the presence of various catalytic amounts of UoB-20 in different solvents and at diverse temperatures to provide benzaldehyde.

**Fig. 6:**
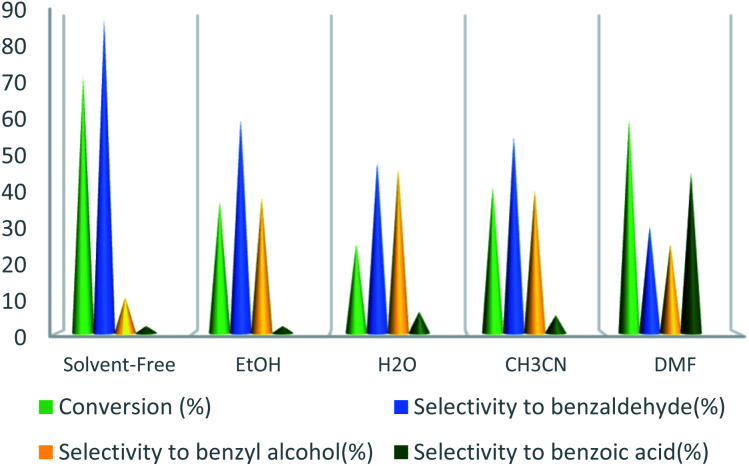
Effect of solvent on toluene oxidation. Reaction condition toluene: 1 mmol; UoB-20: 2 mol%; toluene/TBHP mole ratio: 1/3; time: 90 min, temperature: 75 °C.

At first, according to reported articles^30–32^ the polar solvents, *e.g.*, H_2_O, EtOH, and CH_3_CN were selected as the reaction solvents at room temperature, but no product was achieved. In the next step, it was decided not to use any solvent that can be used at a higher temperature without the requirement of a reflux condenser. However, no product was achieved again from performing the reaction in solvent-free conditions at room temperature. To improve the reaction conditions, the temperature was set at 75 °C in a solvent-free medium. At this time, the desirable product was gained with a good yield. Next, other solvents were used instead of the solvent-free condition and the reaction repeated in the presence of UoB-20 (2 mol%) at 75 °C for 90 minutes (Fig. 6). The toluene conversion was increased in a weak polar solvent (such as DMF), while selectivity was drastic. Whereas, benzaldehyde as the main oxidation product was obtained in strong polar solvent like water, but with the little conversion. Actually, the significant conversion and selectivity were achieved in a solvent-free medium.

**Fig. 7:**
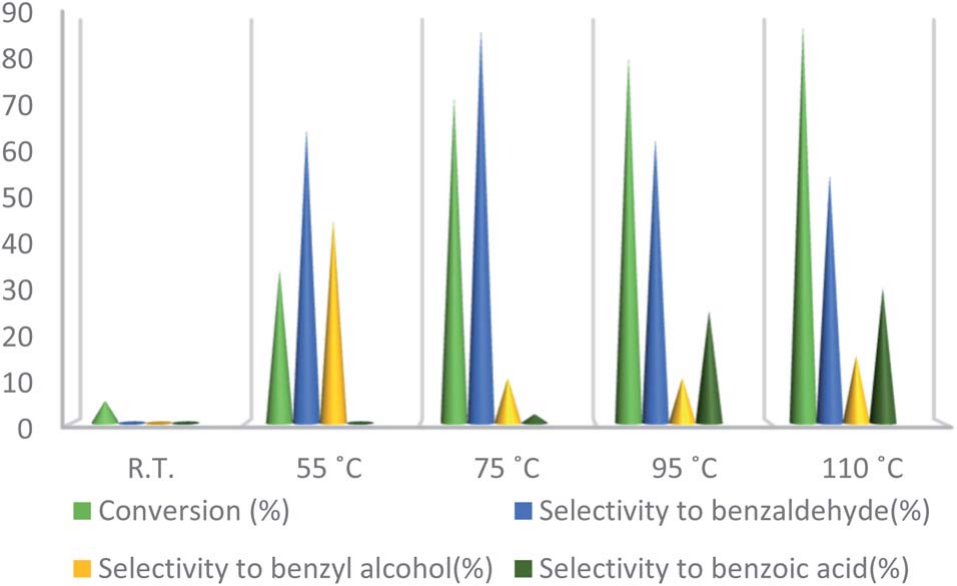
Effect of temperature on toluene oxidation. Reaction condition toluene: 1 mmol; UoB-20: 2 mol%; toluene/TBHP mole ratio: 1/3; time: 90 min.

**Fig. 8:**
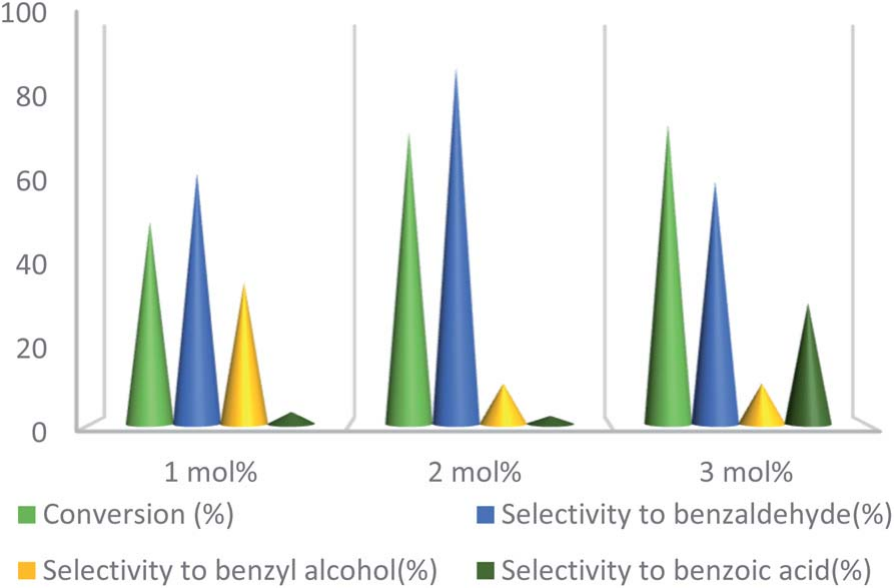
Effect of catalyst amount on toluene oxidation. Reaction condition toluene: 1 mmol; toluene/TBHP mole ratio: 1/3; time: 90 min, temperature: 75 °C.

**Fig. 9:**
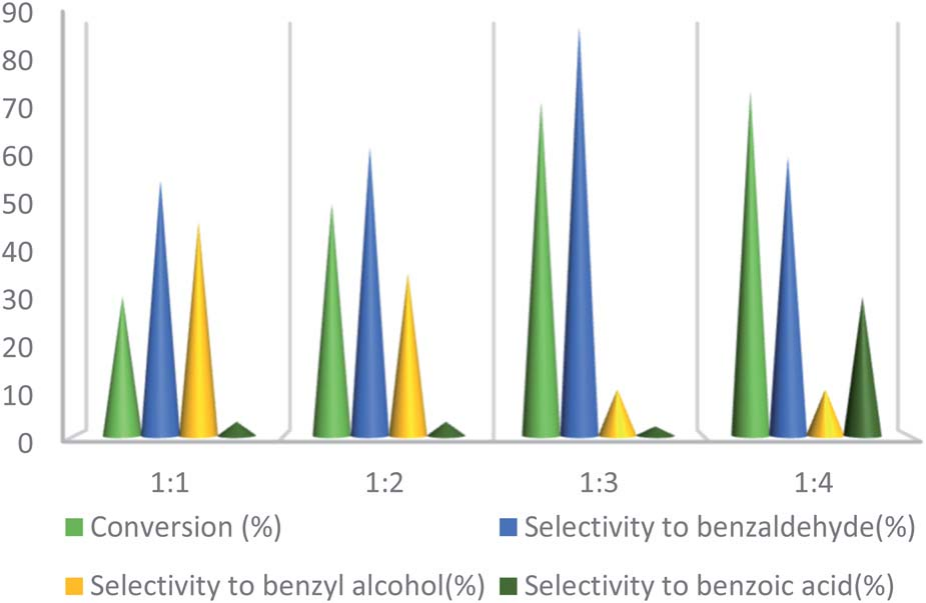
Effect of the molar ratio effect of toluene/TBHP on toluene oxidation. Reaction condition toluene: 1 mmol; UoB-20: 2 mol%; time: 90 min, temperature: 75 °C.

To further study the effect of temperature, the toluene oxidation was investigated at various temperatures (55, 75, 95, and 110 °C). It is obvious that the conversion of toluene increased from 72% to 88% while the reaction temperature increased from 75 °C to 110 °C, while the benzaldehyde selectivity decreased from 87% to 55%. The good conversion of toluene at 75 °C can be due to the thermal effect of the kinetically controlled reaction. So, the conversion of toluene increased as the reaction temperature rose. It should be considered that the reaction selectivity was decreased and more by-products produce with increasing temperature. It could be due to over oxidation of benzaldehyde or the decomposition of TBHP in higher temperature (Fig. 7). Another important factor, which influences the oxidation of toluene, is the catalyst amount (Fig. 8). Toluene conversion increased from 50% to 72%, with an increase in the catalyst amount from 1 mol% to 2 mol%. Whereas, no remarkable change was observed in the toluene conversion by taking 3 mol% of UoB-20. Although, the selectivity was decreased due to occurring over-oxidation along with increasing catalyst amount. The effect of toluene/TBHP molar ratio with respect to time on the oxidation of toluene was also investigated under the condition which other parameters were retained constant (Fig. 9). With increasing the molar ratio of toluene to TBHP from 1 : 2 to 1 : 3, the toluene oxidation was increased. It may be due to the release of the high amount of oxygen on the decomposition of TBHP, which is responsible for the toluene oxidation. While an excessive amount of released oxygen is the favour for over-oxidation and the formation of benzoic acid.

It is necessary to mention that H_2_O_2_ was also used as an oxidant in the toluene oxidation, and TBHP exhibited a higher activity than H_2_O_2_. In the oxidation of toluene with H_2_O_2_ as an oxidant, carboxylic acid was produced as a by-product, but no by-product was gained in the presence of TBHP.

**Fig. 10:**
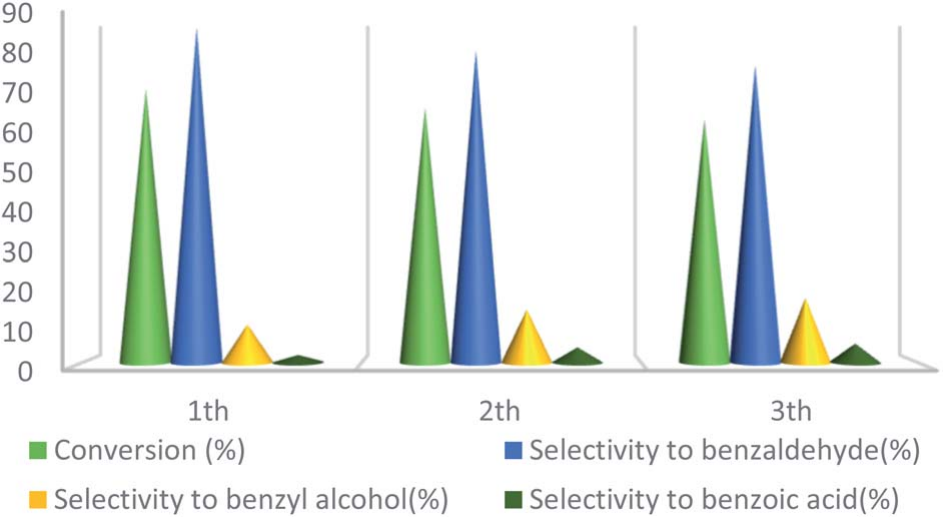
Recyclability tests of UoB-20 for the oxidation reaction of toluene to benzaldehyde.

UoB-20 was also examined for reusability in the oxidation of toluene at the optimized reaction conditions (Fig. 10). After completion of each reaction, the catalyst was separated by centrifuge, washed with ethanol, dried at 80 °C for overnight, and employed in the next run. The toluene conversion decreased from 72% to 64% and selectivity of benzaldehyde decreased from 88% to 78% after the 3rd cycle. The decreasing the catalytic activity in the subsequent run may be due to the partial filling porous during the reaction.

**Scheme 3:**
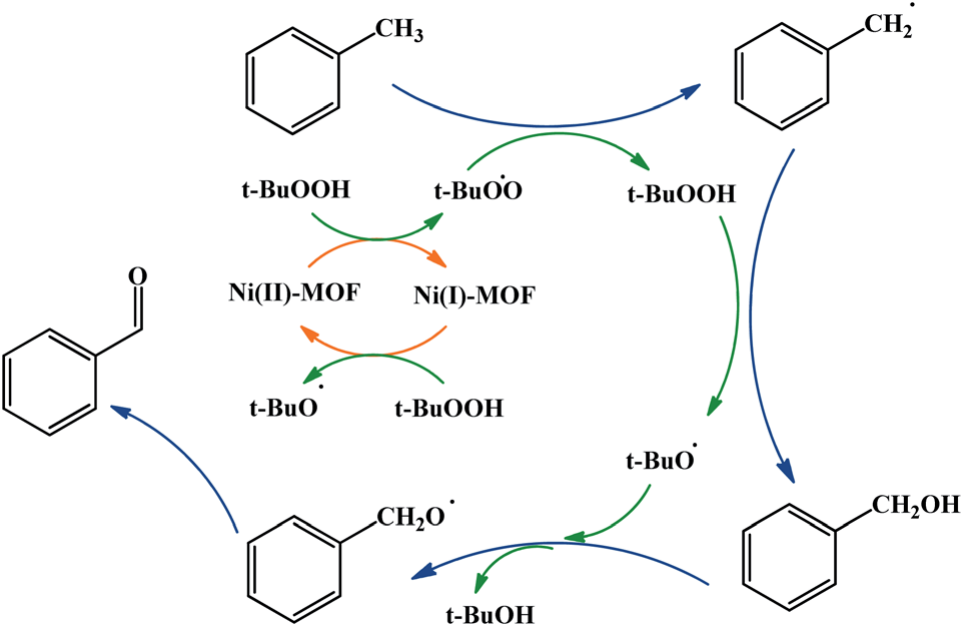
Proposed mechanism for the effect of Ni-MOF and TBHP in toluene oxidative reaction.

A possible mechanism for toluene oxidation by TBHP in the present of UoB-20 was proposed and is depicted in Scheme 3. It should initially involve the redox reactions of the oxidant TBHP by Ni(ii)/Ni(i) metal centers and the formation of oxygen-based radicals of *t*-BuOO˙/*t*-BuO˙ nickel(ii) firstly turned into nickel(i) with donating an electron to TBHP and forming *t*-BuOO˙. Then, nickel(i) turned into nickel(ii) along with the generation of *t*-BuO˙ with absorbing an electron from a molecule of TBHP. Next, *t*-BuOO˙ with abstracting a hydrogen atom from toluene formed the radical intermediate, that turned into alcohol with another TBHP. In continuation, a second radical intermediate was provided with coupling *t*-BuO˙. This intermediate turned into benzaldehyde with adsorbing an electron. To confirm the path of the radical mechanism, the toluene oxidation was catalysed by UoB-20 in presence of CBrCl_3_ and PhNH_2_ (well-known carbon–oxygen or -radical traps,^33^ respectively). When CBrCl_3_ and PhNH_2_ were added to the reaction mixture, the formation of benzaldehyde was not occurred. This observation revealed the involvement of radical species in the oxidation mechanism and it is similar to the proposed mechanism in other cases.^34^ So, it can be concluded that the mechanism involve the metal-assisted generation of *t*-BuOO˙ and *t*-BuO˙ radicals upon redox reaction of *t*-BuOOH.

## Conclusions

The novel Ni-MOF was successfully synthesized using H_3_TATB (4,4′,4′′-*s*-triazine-2,4,6-triyl-tribenzoic acid), as a linker containing imine groups, and confirmed by various characterization techniques. The CO_2_ adsorption capacity for this framework was studied that revealed relatively significant capacity under flue gas conditions. The results of comparison Ni-MOF with other MOFs for CO_2_ adsorption capacities exhibited that Ni-MOF has a good activity despite lower surface area respect to reported previous MOFs. In addition, the prepared Ni-MOF was used in the toluene selective oxidation, which showed excellent catalytic activity and high selectivity for producing benzaldehyde. However, the catalyst was simply recovered and reused for three times without evident activity loss. In addition to the results reported above, this paper could provide insight into developing metal–organic frameworks with enhanced activity in various aspects.

## Conflicts of interest

There are no conflicts to declare.

## Supplementary Material

RA-010-D0RA05233G-s001
