# Supplementary material for: Investigation of the carbon dioxide adsorption behavior and the heterogeneous catalytic efficiency of a novel Ni-MOF with nitrogen-rich channels
Source: RSC Adv. 2020 Aug 12;10(50):29772–9. doi: 10.1039/d0ra05233g (PMC9056285; doi:10.1039/d0ra05233g)
Supplement: RA-010-D0RA05233G-s001 [file RA-010-D0RA05233G-s001.pdf]

Electronic Supplementary Material (ESI) for RSC Advances.
This journal is © The Royal Society of Chemistry 2020

Investigation of the carbon dioxide sorption behavior and heterogeneous catalytic efficiency by a new Ni-MOF with nitrogen-rich channels

Sima Aryanejad [a], Naser Valipour Motlagh [a1]

[a] Department of Chemistry, Faculty of Science, University of Birjand, Birjand, Iran

Synthesis of 4,4′,4″-s-triazine-2,4,6-triyl-tribenzoic acid (H_3TATB)

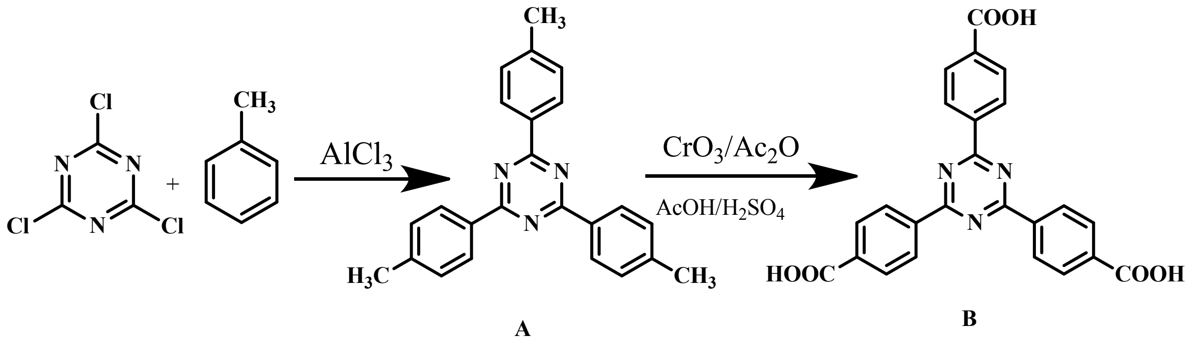

Scheme S1: Synthetic procedures of H3TATB

Synthesis of A:

$AlCl_3$ (20 g) was added into a 250 ml three-connected flask containing dry toluene (50 ml). $C_3N_3Cl_3$ (8.3 g) was then added gradually at 60 ˚C. After that, the mixture was stirred overnight. To quenching catalyst, the resulting red sticky oil was poured into a large amount of ice water. The water layer was decanted by adding $CHCl_3$ (100 mL), and the organic layer was filtered. Methanol was added into $CHCl_3$ to precipitate some needle-like solid. The rest of the solid was dissolved in hot toluene, then place in refrigerator to afford white needle-like crystalline solids. ^{1}H NMR (δ, $CDCl_3$): 2.46 (s, 9 H), 7.35 (d, 6 H), 8.64 (d, 6H).

Synthesis of H_3TATB:

Compound C (2.78 g) was dissolved in acetic acid (70 mL), and H_2SO_4 (4.4 mL) was added it. Chromium oxide (7.2 g) was dissolved in acetic anhydride (4.8 mL) with stirring, which was then added into the previous mixture slowly, using a cold water ice bath to keeping the temperature below 50 ˚C. The black-brown slurry mixture was stirred overnight. After that, the resulting was poured into cold water (300 mL), stirred for 1h, and then filtered. For remove

* Corresponding author: n.valipour.m@birjand.ac.ir, nasservalipoormotlagh@gmail.com,
Tell: 0989153636841, Fax: 098-5632202515

chromium acid, the solid was washed with water. Then, the white precipitate was dissolved in NaOH solution (200 mL, 2N). The unreacted starting material was removed by filtration, the solution was acidified (pH<3) with HCl solution (10%) to give white crude product precipitate. The final product was filtered and dried in an oven. Recrystallization of the product with DMF gave the pure product as a white solid. FTIR (cm^{-1}): 3115, 2770, 2474, 1716, 1490, 1405, 1065, 809, 768. ^{1}H NMR (400 MHz, d_6-DMSO, δ): 7.96 (d, 6 H), 8.41 (d, 6 H), 13.13 (br, 3H). ^{13}C NMR (100 MHz, d_6-DMSO, δ): 128.85, 129.91, 134.87, 138.74, 167.13, 170.45.

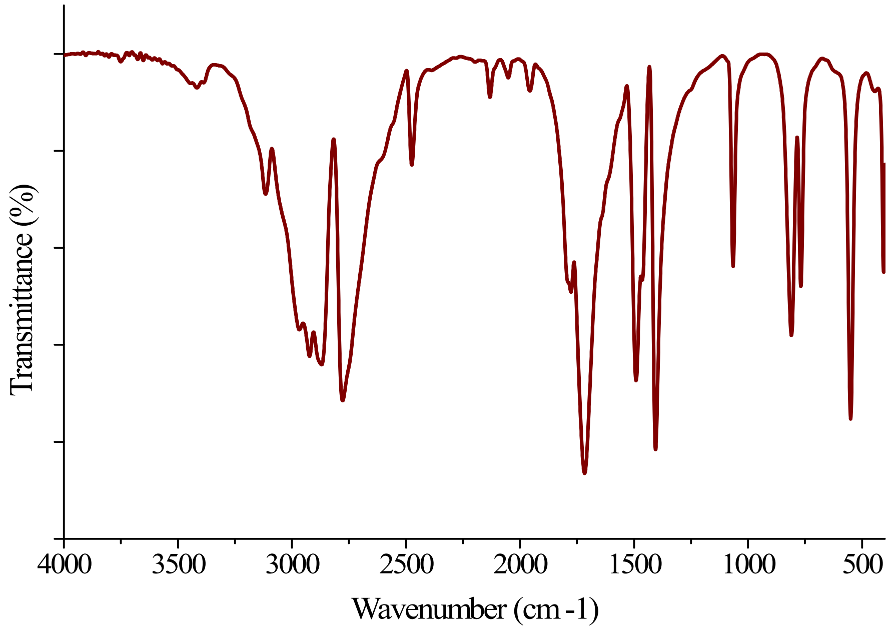

Figure S1: FT-IR spectrum of H_3TATB

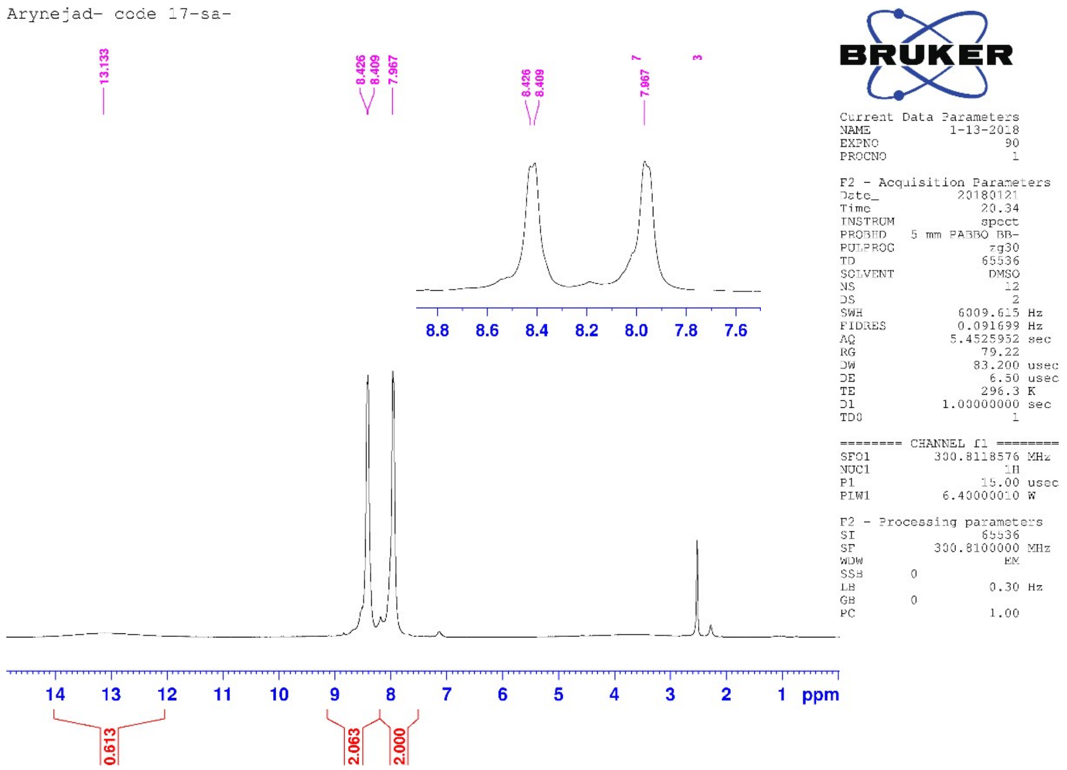

Figure S2: ^{1}H-NMR of H_3TATB

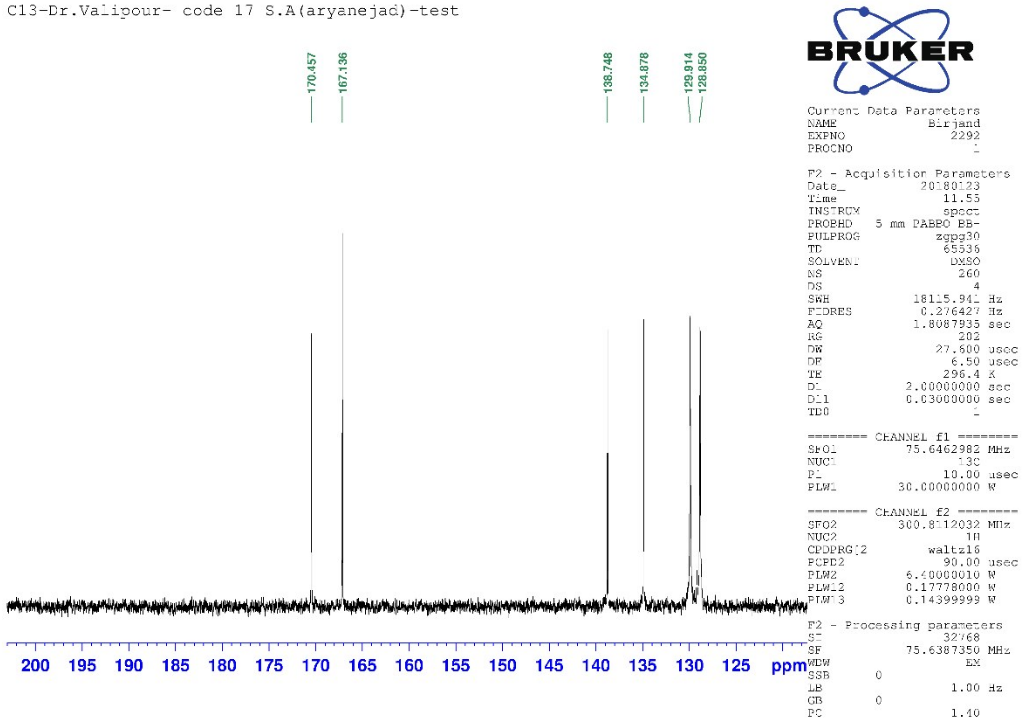

Figure S3: ^{13}C-NMR of H_3TATB
